# Mediæval Remedies for the Plague.—III

**Published:** 1898-07-02

**Authors:** 


### Modern Sociology.

### MEDIÆVAL REMEDIES FOR THE PLAGUE.—III.

At every street corner the quack, both male and female, drove a roaring trade. The composition of the lozenges, electuaries, and waters sold as infallible cures is all unknown. Times have altered little in that respect, and impudence in advertising was all that was needed to provoke a ready sale. The most highly esteemed preparations were those in which gold was reputed to form an ingredient, and a recipe for the home-brewing of one such "sovereign drink" has survived. "Take a piece of fine gold, or the leaves of pure beaten gold, and put it into the juice of lemons, and let it lie therein twenty-four hours; add a little powder of angelica roots and mingle with white wine; this is most precious, and has recovered many past all hope." Something of this nature, it may be believed, was an antidote called aurum vitæ, invented and produced in 1640, by John Woodall, chirurgeon of the East India Company, and of His Majesty's Hospital of Saint Bartholomew in London. The drug was administered in the form of a tiny powder, mixed with a little mithridatum or nap of apple, or drunk with a posset of ale and milk. A remarkable testimony to its wide-spread popularity among influential persons is afforded by a curious document printed at the end of Woodall's pamphlet, and purporting to be a copy of a "certificate concerning the virtues of the antidote called aurum vitæ, from the justices, ministers, and other officers of the parish of St. Margaret Westminster, presented to Right Honourable Henry Earl of Manchester, Lord Privy Seal." In this certificate the wonderful effects of the powder are detailed with a final declaration, attested by a long list of names, that "none died who took it."

Among the more peculiar prescriptions for the plague-stricken it is impossible to omit those of Leonardo Fioravanti, translated by Thomas Hill, with a dedication to Sir James Blount by no less a person than the eminent surgeon John Hester, attached to the person of Queen Elizabeth.

Fioravanti's first suggestion for those sickening with the plague, "to stand in or bathe in the waters of the sea four or five, or ten or twelve hours," is sufficiently startling, especially as it appears he means actually the open sea, and the translator has overlooked the fact that our northern waters would render the treatment even more drastic than Fioravanti contemplated. But this remedy is mildness itself compared with that which follows: "When a man have a pestilent sore, let there be made a hole in the earth, and there let him be buried all save neck and head, and there let him stand fifteen or eighteen hours, and he shall be holpen and then take him forth. The earth is our mother, and purifieth all things." He argues in favour of this treatment from his experience of the benefit afforded to many by the mud baths at Bologna, but refrains from mentioning whether he had ever enjoyed an opportunity of putting the experiment to proof. He does not, however, doubt of its commending itself to many, and concludes with the unanswerable argument that it is both "cheap and easy."

